# Strategies Following Free Flap Failure in Lower Extremity Trauma: A Systematic Review

**DOI:** 10.1016/j.jpra.2023.03.002

**Published:** 2023-03-29

**Authors:** Isabelle T.S. Koster, Marieke P. Borgdorff, Faridi S. Jamaludin, Tim de Jong, Matthijs Botman, Caroline Driessen

**Affiliations:** a Department of Plastic Reconstructive and Hand Surgery, Amsterdam University Medical Center, VU Amsterdam/University of Amsterdam, Netherlands; b Department of Plastic Reconstructive and Hand Surgery, Radboudumc, Nijmegen, Netherlands; c Amsterdam UMC location University of Amsterdam, Medical Library AMC, Amsterdam, Netherlands

**Keywords:** Lower extremity, Traumatic injuries, Free flap, Flap failure, Microsurgery, Treatment outcome

## Abstract

**Background:**

Free flap reconstructions are an important reconstructive option for soft tissue defects in mangled lower extremities. Microsurgery facilitates soft tissue coverage of defects that otherwise would result in amputation. However, the success rates of traumatic lower extremity free flap reconstructions remain lower than those in other locations. Nevertheless, post-free flap failure salvage strategies have rarely been addressed. Therefore, the current review aims to provide an overview of post-free flap failure strategies in lower extremity trauma and their subsequent outcomes.

**Methods:**

A search of Pubmed, Cochrane, and Embase databases was performed on June 9, June 2021 using the following medical subject headings (MeSH) search terms: ‘lower extremity’, ‘leg injuries’, ‘reconstructive surgical procedures’, ‘reoperation’, ‘microsurgery’ and ‘treatment failure’. This review was conducted in accordance with Preferred Reporting Items for Systematic Reviews and Meta-Analyses (PRISMA) guidelines. Partial and total free flap failures after traumatic reconstruction were included.

**Results:**

Twenty-eight studies with a total of 102 free flap failures fulfilled the eligibility criteria. Following the total failure, a second free flap is the predominant reconstructive strategy (69%). In comparison to the failure rate of a first free flap (10%), the fate of a second free flap is less favorable with a failure rate of 17%. The amputation rate following flap failure is 12%. The risk of amputation increases between primary and secondary free flap failures. After partial flap loss, the preferred strategy is a split skin graft (50%).

**Conclusion:**

To our knowledge, this is the first systematic review on the outcome of salvage strategies after free flap failure in traumatic lower extremity reconstruction. This review provides valuable evidence to take into consideration in the decision-making regarding post-free flap failure strategies.

## Introduction

**Figure 1:**
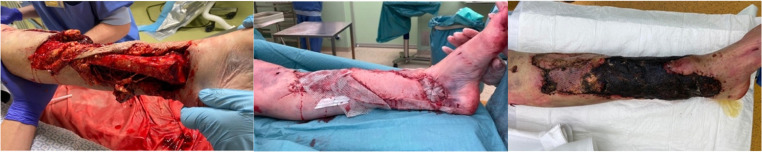
Left: Patient P (51/f) suffering a high-energy trauma resulting in a lower extremity Gustilo 3b injury. Middle: the patient received a myocutaneous latissimus dorsi free with an end-to-end anastomosis to the tibial artery and vein 10 days after trauma. Right: seven days after placement total free flap failure, resulting in complete removal and despite negative pressure wound therapy eventually amputation.

The treatment of lower extremity trauma and the vast impact it wields on patients’ lives has remained a topic of research for decades.^1^ Free tissue transfer is the most sophisticated solution for wound closure^2^^,^^3^ yet still traumatic lower extremity wounds are among the most complicated defects to repair.^4^, ^5^, ^6^ Free flap success rates remain comparatively lower than other wound etiologies and defect locations.^7^ Overall, free flap success rates reside at more than 95%,^8^, ^9^, ^10^ whereas extremity reconstruction lags at as low as 80%.^8^^,^^11^^,^^12^ Due to their complicated nature, free flaps for lower extremity trauma historically display an increased chance of complications, where even partial flap loss can result in total reconstructive failure. The failure of these free flaps, as shown in Figure 1, contributes to a considerable portion of post-traumatic morbidity and can result in limb amputation,^11^^,^^13^, ^14^, ^15^ impaired functional recovery,^16^ substantial healthcare costs,^17^^,^^18^ and consequently a lower quality of life for the patient.^10^^,^^15^ Thus, when a free flap fails, an adequate reconstructive strategy must be in place to avert the potentially detrimental consequences.

Post-free flap failure strategies have rarely been addressed in the literature. In 2010 Lineaweaver and colleagues published a review summarizing free flap failures across all body regions.^3^ Similarly, studies have compiled free flaps for a broad range of injury etiologies^19^, ^20^, ^21^, ^22^ and recorded the outcomes following free flap failure. These studies have effectively identified the range of potential treatments following failure, including the application of a second free flap and also less complex solutions such as local flaps, negative pressure therapy, skin grafts, wound dressing, or amputation. Nevertheless, a focused review of the strategies after traumatic free flap reconstruction failure in lower extremities is lacking to date.

The current review aims to provide an overview of post-free flap failure strategies in lower extremity trauma and their subsequent outcomes. This concise overview of existing data will contribute to more efficient and successful decision-making in the future.

## Methods

This systematic review was conducted according to the Preferred Reporting Items for Systematic Reviews and Meta-analyses (PRISMA) reporting guidelines. Details of the protocol were registered at PROSPERO, the International prospective register of systematic reviews (PROSPERO; CRD42022296979).^23^

**Figure 2:**
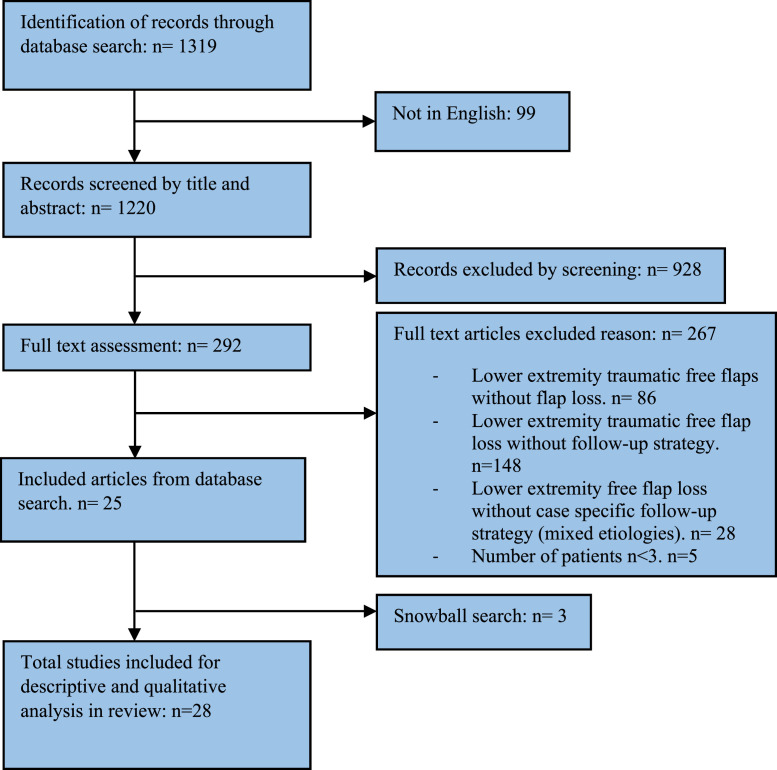
PRISMA flow chart of search and selection.

A Pubmed, Cochrane, and Embase search was performed on the 9th of June 2021 using the following MeSH search terms: ‘lower extremity’, ‘leg injuries’, ‘reconstructive surgical procedures’, ‘reoperation’, ‘microsurgery’ and ‘treatment failure’ (Supplementary material 1). This search returned an unduplicated result of in total of 1319 articles. Authors IK and MB independently screened the titles and abstracts of the 1319 search results using the online available research tool Rayyan.^24^ Following this, full-text articles were reviewed. Any discrepancies were resolved through discussion and if necessary, by the senior author (CD) for reaching consensus. The selection process is depicted in Figure 2.

Prospective or retrospective studies describing patients of all ages with failed free flaps after lower extremity trauma were eligible for inclusion and therefore read fully. Non-English language articles were not considered for inclusion. No publication date restrictions were applied. All types of publications were eligible, except for case reports, letters, editorials, or comments on other existing articles. Both partial and total free flap failure were deemed relevant for analysis. Partial failure is described as the loss of a part of the free flap substantial enough to compromise its function for wound closure, thus requiring further intervention.^20^^,^^21^ The free flaps were permitted to be of any configuration (fasciocutaneous, myocutaneous, muscular, osteocutaneous) except for buried bone flaps without the possibility of flap monitoring.

Studies including failed traumatic lower extremity-free flaps *without* a clearly described case-by-case follow-up procedure were excluded. Studies with a mixture of free flap indications with unspecific mentions of follow-up strategy were excluded on the grounds of lack of detail. Studies with patients undergoing reconstructions for atraumatic etiologies were also excluded. Studies with three patients or fewer were considered case reports and therefore not included. Authors of studies that described relevant data but were not case specific were contacted by email for clarification of results, a successful endeavor for the inclusion of one article.^22^

Data extraction was performed by investigator IK and confirmed by MB. The extracted data from all studies included: study design, country of origin, study timeframe, total traumatic free flaps performed in the study, total failed traumatic free flaps, and follow-up time. All failed free flaps were specified according to the patient's age, sex, defect location, mechanism of injury, time to free flap, donor site of free flap, anastomosis, partial or total failure, cause of failure, follow-up procedure, time until secondary procedure and outcome after secondary procedure. Comorbidities were often not mentioned and therefore not considered in the results of the current study. The primary outcome was that the strategy employed post-free flap failure. The secondary outcome was the subsequent outcome of that management strategy. Additionally, etiologies of failed flaps other than acute trauma were included when directly linked to a traumatic origin, such as post-traumatic osteomyelitis or post-traumatic pseudoarthrosis.

All included studies were rated according to the Oxford Centre for Evidence-Based Medicine (CEBM) level of evidence scale by the primary and secondary authors in a blinded format. A meta-analysis was not deemed feasible due to the heterogeneity of data in our collected studies. Instead, a descriptive and qualitative analysis was performed.

The methodological quality was assessed using the Study Quality Assessment Tool developed by the National Institutes of Health.^25^ First and second authors independently assessed the quality of included articles. Studies were deemed adequate for inclusion with ratings of either ‘fair’ or ‘good’ (Supplementary material 2).

## Results

### Study and patient characteristics

A total of 28 studies met the inclusion criteria. Of these studies, 27 were retrospective and one was prospective. Across the studies, more than 3000 microsurgical free flaps were performed of which 1020 free flaps were post-traumatic lower extremity free flaps. Out of these lower extremity-free flaps, 102 losses were recorded, representing an overall flap failure rate of 10% (4.7% partial, 5.3% total). A summary of all cases is presented in Table 1. Of the failed flaps where gender was documented, 52 were male (80%) and 13 were female (20%). Patient and free flap characteristics are summarized in Table 2. Importantly, all studies described a case-by-case strategy after failure. Twenty out of the 28 studies (71%) additionally described the outcomes of the strategies. Two studies were multicenter studies. The study timeframe ranged from 1 to 22 years. Seven studies were cohort studies, whereas the remaining majority were case series that were identified out of larger populations.

**Table 1:** Summary of included studies and the number of (failed) free flaps in each study.

	No. of flaps performed in study	Number of LE trauma free flaps	Total failed free flaps (all etiologies)	Failed LE traumatic free- flaps
Arslan, 2012^26^	18	18	2	2
Baumeister, 2008^27^	902	8	13	8
Chiang, 1997^28^	25	25	3	3
Egozi, 2011^29^	9	4	4	3
Fearon, 1990^30^	300 (+)	5	7	5
Hallock, 2013^22^	310	4	6	4
Hallock, 2014^31^	14	1	1	1
Hallock, 2014^32^	314	5	21	5
Hutson, 2010^33^	18	18	3	3
Irons, 1983^34^	15		4	2
Khoo, 1982^35^	4	4	3	3
Kim, 2016^36^	16	16	4	4
Kim, 2019^37^	16	16	3	3
Kolker, 1997^38^	451	451	30	3
Koski, 2004^39^	35	35	2	2
Lin, 2004^40^	65	48	10	5
Lowenberg, 2015^41^	127	127	6	6
Luangjarmekorn, 2017^42^	35	13	13	7
Messner, 2020^15^	17	17	3	3
Ozkan, 2016^43^	8	7	1	1
Repo, 2016^44^	13	4	4	4
Seo, 2018^45^	5	3	5	3
Smit, 2012^6^	16	13	1	1
Top, 2006^46^	13	1	4	1
Ulusal, 2005^5^	50	32	4	2
Vaienti, 2013^47^	4	4	4	1
Weinzweig, 1995^48^	140		10	3
Yalcin, 2021^49^	141	141	14	14
Total	3081	1020	185	102

**Table 2:** Patient characteristics, failed free flap etiologies, and primary free flap type.

	No. of cases
**Gender**	
Male	52
Female	13
Unreported	37
**Age**	4-76
**Follow up time**	4mo-11y
**Indication for reconstruction**	
MVA	10
Pedestrian vs car	7
Fall from height	3
Gunshot	3
Machinery	3
Compartment syndrome	1
Other	13
Unspecified	62
**Type of free flap**	
Latissimus dorsi	28
Anterolateral thigh	11
Ilium/iliac crest	8
Gracilis	6
Free fibula	6
Thoracodorsal artery perforator	4
Lateral arm flap	3
Groin flap	3
SCIP	2
Rectus abdominis	2
Medial sural artery perforator	2
Omentum	2
Parascapular	1
Scapular	1
Sural	1
Double free flap	4
Free radial forearm	1
Unreported	17
**Gustilo**	
IIIB	12
IIIC	1
Unknown	89
**Arterial anastomosis**	
Tibial artery	31
Other: PeA, SFA, PoA, SA	10
Unreported	61
End-to-end	12
End-to-side	14
Unreported	76

MVA: motor vehicle accident; PeA: peroneal artery; SFA: superficial femoral artery; PoA: popliteal artery; SA: sural artery

Eighty-nine out of the 102 failure cases were deemed failures after primary traumatic reconstructions. The remaining 13 were cases of secondary post-traumatic reconstructions, which are described in Table 2 as the category ‘other’. These post-traumatic injuries included post-traumatic osteomyelitis (7 cases), a post-traumatic deep infection (1), a post-traumatic severely unstable scar (1), post-traumatic ulceration (1), post-traumatic malunion (1), ulceration (1) and post-traumatic pseudoarthrosis (1). Oftentimes, the zone of injury was described as the lower leg (28%) or tibia (27%), without further specification.

Timing of the free flap reconstruction after trauma was recorded in 62 patients (61%). Of these recorded failed cases, 6.5% were acute reconstructions (<72 hours), 39% were subacute reconstructions (<90 days), and 55% were delayed reconstructions (>90 days). The time between failure and second reconstruction was recorded in 25 patients (25%); 72% of these patients had reconstruction in the subacute period; 24% in the delayed period, and 4% in the acute period. In 41 patients, information was provided concerning arterial anastomosis (Table 2). The tibial artery was most often reported as being used for arterial anastomosis. Venous anastomosis was described in 32 patients; of which 28 cases (88%) were using the concomitant vein. In general, anastomotic data was scarce. Little to no information was available on the use of venous couplers or the number of veins utilized in the anastomoses.

### Flap failure and subsequent strategy

Fifty-three percent of all failures were total flap failures. In the total flap failure group, venous thrombosis (28%) followed by infection (20%) and arterial thrombosis (19%) were the most commonly reported causes of failure (Table 3). Venous thrombosis was also most often the cause of partial failure (22%), followed by infection (13%). Total failure was predominantly remedied by a second free flap (69%) followed by amputation (15%). Flap failure was deemed to be a partial failure in 47% of cases, in which split skin grafts were most commonly employed (50%), followed by a second free flap (19%). The post-failure strategies of all 102 failed lower extremity-free flaps are summarized in Table 4. Overall, the most common management strategy across all studies employed a new free flap (45%). However, in 41 cases (40%) no outcome of these strategies was mentioned.

**Table 3:** Reported causes for free flap failure.

Reported cause of failure	Total No. of cases	Flap loss	
		Partial failure N= 48	Total failure N= 54
Venous thrombosis	26	11	15
Infection	17	6	11
Arterial thrombosis	14	4	10
V + A thrombosis	8	5	3
Hematoma	4	4	
Arterial spasm	2	1	1
Traumatic injury to flap	2		2
Anastomotic failure	2		2
Hemorrhage	1	1	
Unknown	5		5
Not reported	21	16	5

**Table 4:** Strategies following free flap failure and outcomes of strategies.

Follow-up strategy	No. of cases total N=102	Flap loss		Follow-up strategy outcome			
		Partial N=48	Total N=54	Uncomplicated	Failure	Unreported	Complication
Second free flap	46	9	37	27	8	9	2
Split skin graft	28	24	4	11	1	16	
Amputation	8	0	8	2		6	
Local flap	7	5	2	2		5	
Healing by secondary intention	5	4	1	2		3	
VAC + SSG	4	4	0	3		1	
VAC + local flap	2	2	0	2			
VAC + conservative healing	2	0	2	1		1	

The fate of a second free flap is less favorable demonstrating a failure rate of at least 8 out of 46 (17%) versus 10% in a first free flap. In these eight cases of failed *second* free flaps, only one case received a successful third free flap, three did not receive a new free flap but instead were left to heal by secondary intention, and four resulted in limb amputation. One out of 28 (3.6%) cases split skin grafts following failed initial free flap ended in graft loss, partial failure of the free flap, and the need for a new successful free flap.^35^ All other post-free flap failure strategies including local flaps, healing by secondary intention, or negative pressure therapy in combination with any of these tactics, did not report the need for follow-up procedures. Across all failed free flaps (eight following the first free flap and four following a second free flap), 12 patients (12%) underwent an amputation because of failure of free flap reconstruction.

## Discussion

The current review provides an overview of the outcomes of post-flap failure strategies in traumatic lower extremity reconstructions. The cumulative failure rate of 10% across all our included studies is slightly lower than often mentioned but echoes the trends across existing literature for lower extremity trauma-free flaps.^7^^,^^19^^,^^20^^,^^50^ The majority of patients with total flap failures (69%) undergo a second free flap. This review shows that in comparison to the first free flap (90% success rate), the fate of a second free flap is less favorable (82% success rate). The amputation rate across all studies after free flap failure was 12%, including four cases of amputation following a second free flap failure. Amputation rates vary considerably when examining past studies, from 3.4%^9^ to 22%.^51^

Importantly, the overall data show that repetition of a free flap is the most popular strategy after the total loss of a free flap. A local non-microsurgical reconstruction may not suffice, making a new free flap the only viable option (save for amputation). For partial flap loss, a split skin graft should be considered, with a high overall success rate (only a single failure reported). Similarly, Lineaweaver and colleagues reported that split skin grafts were most commonly turned to as a post-failure strategy in both breast and head and neck reconstructions.^3^

Vascular insufficiency is a leading cause of flap failure in traumatic lower extremity-free flap failure throughout time.^19^^,^^20^^,^^52^, ^53^, ^54^ In this review, venous thrombosis was the most common recorded cause of failure across the studies. Combining this with the cases of arterial and total pedicle thrombosis accounts for the majority of recorded causes in the included studies. In lower extremity trauma, both the artery and the vein may be damaged by the crush of the trauma. Preoperative (CT or real-time) angiography provides important information on the patency of the artery yet lacks sensitivity to find subtle changes to the intima layer. Alternatively, the veins can be studied individually through duplex ultrasound but this is not always possible in an injured leg. Notably, poly-trauma patients reside in a state of hypercoagulability which poses a challenge to microsurgical reconstructions, to begin with.

In early work led by the Godina paradigm, a traumatic lower extremity reconstruction was believed to best take place in the acute setting (within 72 hours of injury), after which point the risk of flap failure would become significantly higher until 6 weeks after injury.^52^^,^^55^ Recent studies however have proposed that the outcome of lower extremity reconstructions is not as heavily correlated to the timing of the reconstruction as previously thought, allowing for more leniency in the timing of the reconstruction.^50^^,^^56^, ^57^, ^58^, ^59^ In this review, the majority of the reconstructions were carried out in the subacute and delayed period. Further research is needed to determine if waiting may increase the success rate of subsequent free flaps following the failure of the first free flap reconstruction.

The majority of the second free flap reconstructions in the cohort of this review heal uneventfully. However, the second free flap failure rate was 17% suggesting an increased risk of failure compared to a primary free flap. The consequences of the second failure also appear to be more severe with 50% resulting in amputation. This is a novel finding which has not previously been demonstrated in a review. These repeat failures could represent cases in which the vascular trauma of the recipient's vessels extends beyond the primary anastomosis site, as is quite often the case in lower extremity trauma. It is therefore imperative when considering a second free flap as a post-failure strategy, to reassess any patient-related risk factors, the benefits and necessity of a new free flap, as well as the overall post-traumatic angiographic status. Furthermore, before submitting the patient to a second free flap, the potentially added donor site morbidity both in terms of form and function versus the added value to the recipient site must be considered as was proven when a second latissimus dorsi flap could result in increased vulnerability of the shoulder.^28^

There are a few limitations to this review. First, due to the lack of uniformity in studies recording post-flap failure strategies, oftentimes important data remains unreported or poorly understood. Several studies presented unspecific patient data which rendered it ineligible for collection. This resulted in the exclusion of several articles with relevant outcomes. In the included studies, comorbidities were inconstantly reported and therefore were not included in the analysis. These comorbidities may contribute to failure and would ideally be available in the future. Prospective, multicentre studies are necessary to interpret and conclude the efficacy of different strategies. Only then will we be able to derive with certainty which strategy has the best chance of success in a particular situation. Second, our data spans four different decades, meaning that the outcomes and management strategies are also a product of the knowledge and developments at the time. This issue further complicates our ability to draw clear-cut conclusions at this time. Finally, we encountered that the definition of partial flap loss was often variable, and we suspect this outcome remains underreported and potentially a more commonly occurring complication than currently recorded.^60^ To date, no report has been published summarizing, and more importantly classifying, what defines partial failure in traumatic free flaps in lower extremities. Though Lie et al*.* studied partial and total failure for the deep inferior epigastric perforator (DIEP) flap breast reconstructions and Knitscke et al. reported different failure categories in the free fibula flap for head and neck reconstruction, grading of the post-traumatic soft-tissue transfer failure is lacking.^60^^,^^61^ Considering these limitations, this review provides a detailed insight into the options if the microsurgical reconstruction of the lower extremity fails. It is suggested that further research should investigate the outcome of post-failure strategies regarding function, patient-reported quality of life, and health-related costs. This will aid in striking a delicate balance of choice between amputation and complex reconstructions.

## Conclusion

This is the first systematic review on the outcome and salvage strategies after free flap failure in post-traumatic lower extremity reconstruction. Flap failure is as common as 10% of microsurgical lower extremity reconstructions in patients treated over the past 40 years across the world. The predominant post-free flap failure strategy in lower extremity trauma is a second free flap, a strategy that is successful in the majority of cases but also proves the chance of flap survival after previous complications are less than in a primary reconstruction. The amputation rate after free flap failure overall is 12%, the risk of which increases between a primary and a secondary free flap.

In conclusion, lower extremity trauma comes with several challenges; occasional flap loss is, at this time, unavoidable. This review provides valuable datasets to take into consideration in the decision-making regarding post-free flap failure strategies.

## Appendix Supplementary materials

Image, application 1
