# Supplementary material for: Strategies Following Free Flap Failure in Lower Extremity Trauma: A Systematic Review
Source: JPRAS Open. 2023 Mar 29;36:94–104. doi: 10.1016/j.jpra.2023.03.002 (PMC10196772; doi:10.1016/j.jpra.2023.03.002)
Supplement: Supplementary file 1 [file mmc1.docx]

**SUPPLEMENTARY MATERIAL**

1. Full search details from all search engines used.

PubMed

737 hits:

("Lower Extremity"[Mesh] OR "Leg Injuries"[Mesh] OR lower extremit*[tiab] OR lower limb*[tiab] OR ankle*[tiab] OR buttock*[tiab] OR foot[tiab] OR feet[tiab] OR hip[tiab] OR hips[tiab] OR leg[tiab] OR legs[tiab] OR knee*[tiab] OR thigh*[tiab])

AND

("Reconstructive Surgical Procedures"[Mesh] OR "Reoperation"[Mesh] OR reconstruct*[tiab] OR reoperat*[tiab])

AND

("Microsurgery"[Mesh] OR microsurg*[tiab] OR micro-surg*[tiab] OR microvascular*[tiab] OR micro-vascular*[tiab] OR free flap*[tiab] OR free vascularized flap*[tiab] OR vascularized free tissue*[tiab] OR free tissue flap*[tiab] OR free tissue transfer*[tiab])

AND

("Treatment Failure"[Mesh] OR fail*[tiab])

NOT

("Letter"[Publication Type] OR "Editorial"[Publication Type] OR "News"[Publication Type] OR "Comment"[Publication Type] OR "Letter"[Title] OR "Editorial"[Title] OR "comment*"[Title])

EMBASE (via OVID):

Database(s): Embase Classic+Embase 1947 to 2021 June 08
Search Strategy:

| **#** | **Searches** | **Results** |
| --- | --- | --- |
| 1 | exp lower limb/ or exp leg injury/ | 626307 |
| 2 | (lower extremit* or lower limb* or ankle* or buttock* or foot or feet or hip or hips or leg or legs or knee* or thigh*).ti,ab,kw. | 887383 |
| 3 | 1 or 2 | 1101458 |
| 4 | reconstructive surgery/ or reoperation/ | 102699 |
| 5 | (reconstruct* or reoperat*).ti,ab,kw. | 448227 |
| 6 | 4 or 5 | 500595 |
| 7 | exp microsurgery/ | 40236 |
| 8 | (microsurg* or micro-surg* or microvascular* or micro-vascular* or vascularized free tissue* or free tissue transfer*).ti,ab,kw. | 128422 |
| 9 | (free adj3 flap*).ti, ab, kw. | 18394 |
| 10 | 7 or 8 or 9 | 153617 |
| 11 | treatment failure/ or graft failure/ | 173722 |
| 12 | fail*.ti,ab,kw. | 1693722 |
| 13 | 11 or 12 | 1745642 |
| 14 | 3 and 6 and 10 and 13 | 1103 |
| 15 | letter/ or editorial/ or note/ or (letter or comment* or editorial).ti. | 2675179 |
| 16 | 14 not 15 | 1095 |

[Cochrane Central Register of Controlled Trials](https://www.cochranelibrary.com/)

Issue 5 of 12, May 2021

ID Search Hits

#1 (lower extremit* or lower limb* or ankle* or buttock* or foot or feet or hip or hips or leg or legs or knee* or thigh*):ti,ab,kw 99682

#2 (reconstruct* or reoperat*):ti,ab,kw 16010

#3 (microsurg* or micro-surg* or microvascular* or micro-vascular* or vascularized free tissue* or free tissue transfer*):ti,ab,kw 5173

#4 (free near/3 flap*):ti,ab,kw 366

#5 #3 or #4 5445

#6 #1 and #2 and #5 52

1. Risk of bias assessment was performed using the Study Quality Assessment Tool developed by the National Institute of Health. Studies were deemed adequate for inclusion with ratings of either ‘fair’ or ‘good’.

| **Author** |  | #1 | #2 | #3 | #4 | #5 | #6 | # | #8 | #9 | #10 | #11 | #12 | #13 | #14 | RoB assessment |
| --- | --- | --- | --- | --- | --- | --- | --- | --- | --- | --- | --- | --- | --- | --- | --- | --- |
| Arslan et al. | case series | Yes | Yes | Yes | Yes | Yes | Yes | Yes | NR | Yes |  |  |  |  |  | Good |
| Baumeister et al. | case series | No | Yes | Yes | Yes | yes | yes | yes | NR | yes |  |  |  |  |  | Good |
| Chiang et al. | case series | Yes | Yes | yes | Yes | yes | no | yes | yes | yes |  |  |  |  |  | Good |
| Egozi et al. | case series | No | Yes | CD | Yes | yes | yes | NR | NA | yes |  |  |  |  |  | Fair |
| Fearon et al. | case series | No | Yes | Yes | *Yes* | Yes | no | NR | NA | Yes |  |  |  |  |  | Fair |
| Hallock 2013 | case series | No | Yes | Yes | no | yes | Yes | NR | NR | Yes |  |  |  |  |  | Fair |
| Hallock 2014a | case series | No | Yes | Yes | yes | yes | yes | NR | NR | yes |  |  |  |  |  | Fair |
| Hallock 2014b | case series | No | Yes | Yes | Yes | yes | yes | NR | NA | yes |  |  |  |  |  | Good |
| Hutson et al. | case series | No | Yes | Yes | Yes | *Yes* | Yes | Yes | NA | Yes |  |  |  |  |  | Good |
| Irons et al. | case series | No | Yes | Yes | Yes | yes | yes | NR | NA | yes |  |  |  |  |  | Fair |
| Khoo | case series | No | Yes | Yes | Yes | Yes | No | Yes | NA | Yes |  |  |  |  |  | Fair |
| Kim et al.2015 | case series | Yes | Yes | Yes | Yes | Yes | yes | yes | NA | yes |  |  |  |  |  | Good |
| Kim et al. | case series | No | Yes | Yes | Yes | Yes | Yes | Yes | NA | yes |  |  |  |  |  | Good |
| Kolker et al | case series | No | yes | Yes | Yes | Yes | Yes | NR | Yes | Yes |  |  |  |  |  | Fair |
| Koski et al. | case series | no | yes | yes | yes | yes | no | yes | NA | yes |  |  |  |  |  | FAIR |
| Lin et al. | cohort | Yes | Yes | Yes | Yes | No | NA | NR | Yes | Yes | No | Yes | No | Yes | No | fair |
| Lowenberg et al. | case series | No | No | Yes | CD | Yes | NR | Yes | NA | Yes |  |  |  |  |  | Fair |
| Luangjarmekorn et al. | cohort | Yes | Yes | Yes | Yes | No | Yes | Yes | NA | Yes | No | NA | NA | yes | No | Fair |
| Messner et al. | cohort | Yes | Yes | Yes | Yes | NA | yes | Yes | NA | Yes | Yes | Yes | NA | Yes | No | Good |
| Ozkan et al. | case series | Yes | Yes | Yes | yes | no | no | NR | NA | yes |  |  |  |  |  | Fair |
| Repo et al. | cohort | Yes | Yes | Yes | Yes | No | Yes | Yes | NA | Yes | NA | Yes | NA | Yes | No | Fair |
| Seo et al. | case series | Yes | Yes | Yes | Yes | Yes | Yes | No | NA | Yes |  |  |  |  |  | Good |
| Smit et al. | case series | yes | yes | yes | yes | yes | yes | yes | NA | yes |  |  |  |  |  |  |
| Top et al. | cohort | Yes | Yes | Yes | Yes | NA | NA | Yes | NA | Yes | NA | Yes | NA | Yes | No | Good |
| Ulusal et al. | case series | No | Yes | CD | No | Yes | Yes | Yes | NA | Yes |  |  |  |  |  | Fair |
| Vaienti et al. | case series | Yes | Yes | NR | Yes | yes | yes | yes | NA | yes |  |  |  |  |  | Good |
| Weinzweig et al. | case series | Yes | Yes | NR | Yes | Yes | No | Yes | NA | Yes |  |  |  |  |  | Fair |
| Yalcin et al. | cohort | Yes | Yes | Yes | Yes | No | NA | NA | NA | Yes | NA | Yes | NA | CD | No | Fair |
